# Colorectal Cancer Pulmonary Metastasectomy: When, Why and How

**DOI:** 10.3390/cancers16071408

**Published:** 2024-04-03

**Authors:** Francesco Petrella, Federica Danuzzo, Maria Chiara Sibilia, Sara Vaquer, Raffaella Longarini, Alessandro Guidi, Federico Raveglia, Lidia Libretti, Emanuele Pirondini, Andrea Cara, Enrico Mario Cassina, Antonio Tuoro, Diego Cortinovis

**Affiliations:** 1 Division of Thoracic Surgery, Fondazione IRCCS San Gerardo dei Tintori, Via GB Pergolesi 33, 20900 Monza, Italy; federica.danuzzo@unimi.it (F.D.); maria.sibilia@unimi.it (M.C.S.); sara.vaquer@unimi.it (S.V.); lidia.libretti@irccs-sangerardo.it (L.L.); emanuele.pirondini@irccs-sangerardo.it (E.P.); andrea.cara@irccs-sangerardo.it (A.C.); enricomario.cassina@irccs-sangerardo.it (E.M.C.); antonio.tuoro@irccs-sangerardo.it (A.T.); 2 Division of Medical Oncology, Fondazione IRCCS San Gerardo dei Tintori, Via GB Pergolesi 33, 20900 Monza, Italy; raffaella.longarini@irccs-sangerardo.it (R.L.); or diegoluigi.cortinovis@irccs-sangerardo.it (D.C.); 3 School of Medicine and Surgery, University of Milano Bicocca, 20126 Milan, Italy

**Keywords:** colorectal cancer, pulmonary metastasectomy, disease free interval, overall survival

## Abstract

**Simple Summary:**

Colorectal cancer (CRC) is the third-most-diagnosed cancer in males and in females, and about 20% of patients diagnosed with CRC present metastatic disease; pulmonary metastasectomy in CRC patients therefore represents a frequent scenario to be managed by thoracic surgeons. Due to the lack of randomized controlled trials among different local strategies, there is no definitive evidence about the optimal approach, although surgical resection is considered the most effective therapeutic option in this clinical scenario.

**Abstract:**

Colorectal cancer is the third-most-diagnosed cancer in males and in females, representing 8% of estimated new cases, and the third cause of cancer-related death in both sexes, accounting for 9% of cancer deaths in men and 8% in women. About 20% of patients diagnosed with CRC present metastatic disease. Although lung metachronous or synchronous metastatic spread without other involved sites has been reported in only a small proportion of patients, considering that this tumor is frequently diagnosed, the clinical approach to CRC pulmonary metastases represents a major issue for thoracic surgeons and CRC oncologists. Among patients diagnosed with pulmonary metastases from CRC, about 9–12% are eligible for local treatments with radical intent, including surgical resection, SBRT (stereotactic body radiation therapy) and ablation therapy. Due to the lack of randomized controlled trials among different local strategies, there is no definitive evidence about the optimal approach, although surgical resection is considered the most effective therapeutic option in this clinical scenario. Oncological achievement of primary radical resection, the biology of primary tumor and metastatic sites, disease free interval and or progression free survival are independent prognostic factors which make it possible to define a cohort of patients which might significantly benefit from pulmonary metastasectomy.

## 1. Introduction

**Figure 1:**
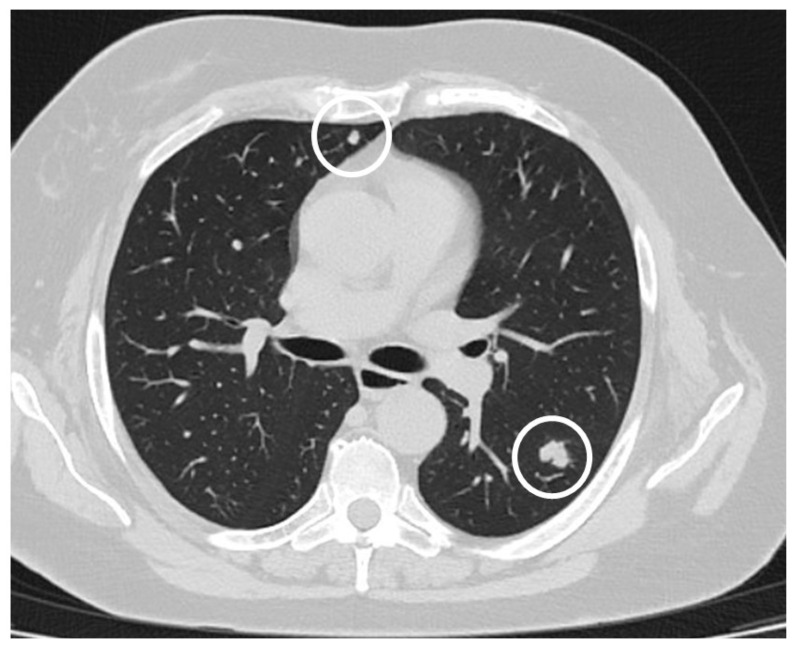
Bilateral lung metastases from colon cancer (white circles), amenable to radical resection by bilateral metastasectomy.

Colorectal cancer (CRC) is the third-most-diagnosed cancer in males and in females, representing 8% of estimated new cases, and the third cause of cancer-related death in both sexes, accounting for 9% of cancer deaths in men and 8% in women [1]. About 20% of patients with CRC present metastatic disease at diagnosis and 40% experience recurrence after treatment of initially localized disease; their prognosis is poor, with a 5-year survival rate of less than 20% having been reported despite the new systemic treatment options [2]. We define as metastatic CRC tumors diagnosed at distant sites after treatment of initially localized disease (metachronous) or CRC already presenting with metastases at first diagnosis (synchronous). The sites most affected by metastases are loco-regional lymph nodes, lung, liver and peritoneum [3]. Among patients diagnosed with CRC, the probability of developing metastases for stage I disease is quite low, with around 10% having been reported; on the contrary, it ranges from 10% to 20% in stage II CRC and from 25% to 50% for stage III disease [4]. Metastatic CRC is defined as resectable when the primary tumor and all metastases can be completely resected; this is usually in the case of a metastastic disease limited to a few target sites—most commonly liver and lung—which can be safely resected by liver and thoracic surgeons in globally fit patients, with a limited amount of healthy parenchyma to be removed [2] (Figure 1). A higher incidence of pulmonary metastases in rectal tumors (5.6%) versus colon tumors (3.7%) has been observed due to the different venous anastomotic circulation that affects the respective anatomical regions. The current approach to CRC is the result of continuous attempts to decrease the incidence and mortality from this tumor: screening programs, systemic treatments in early and surgically resected tumors and aggressive therapeutic approaches represent the most significant efforts to reduce CRC mortality. However, prognosis remains quite poor, particularly in cases of metastatic disease. Although lung metastases have been reported in only a small proportion of patients with CRC, considering that this tumor is very frequently diagnosed, the clinical approach to CRC pulmonary metastases represents a major issue for thoracic surgeons and CRC oncologists. In fact, the clinical practice of lung metastatic involvement in CRC differs from other solid tumors like primary lung cancers or melanoma and renal cell carcinoma (among others), in which the active lung imaging surveillance in standard follow-up is recommended with the specific purpose of diagnosing pulmonary and other metastatic sites in order to decide which locoregional approach is best in the case of oligometastatic disease [5].

## 2. Biology and Physiopathology of CRC Metastases

Within the same primary tumor, there are different cancer cell subpopulations presenting different levels of ability to metastasize, in what is defined as the phenomenon of intratumoral heterogeneity. Metastases development results from selective diffusion of those cells capable of completing all the steps of the multistage metastatic cascade process; this selection occurs in a stochastic way or after biological pressure following the main systemic treatment, such as chemotherapeutic or biological treatments. 

Metastatic tumor cells have to interrupt the basement membrane matrix to penetrate into blood or lymphatic vessels and spread to distant organs. This process may happen by reduction in production or increase in degradation of basement membranes. Once metastatic cells have reached the target organs, they first adhere to the endothelium and then, after extravasation, they invade the parenchyma of the target organs, which are the liver and the lung in the case of CRC. Metastatic CRC cancer cells display an organotropic attitude, the liver and the lung being the most frequent target organs; this is not strictly related to target organ anatomy, blood flow or the total amount of circulating tumor cells spreading to the liver and the lung [6,7]. The lung elective pattern of CRC metatastic spread is not yet completely understood; however, in some clinical trials, the genomic characterization of metastatic sites with respect to primary tumors helps to improve the awareness regarding the association between genomic pattern alterations and metastatic behavior in attempting to classify the organotropic activity of a specific primary tumor [8].

## 3. The History of Metastasectomy in CRC

The idea of additional surgery in already-resected patients presenting cancer recurrence is not new at all, dating back to 1954 when Wangensteen et al. described their experience in 103 patients receiving a “second-look” laparotomy for gastric and CRC cancers diagnosed with lymph nodes metastases at first operation. They re-operated on these patients six months after the first resection and—among sixty-four patients with CRC cancer—they observed a resectable recurrence in 29 cases, which they approached with radical curative intent [9]. On the contrary, some years later, Bacon et al. reported their experience in 93 patients who were re-operated on only in the event of clinical evidence of cancer recurrence: they observed inoperable recurrence in 55 out of 93 reoperated patients [10]. Which approach was to be preferred remained unknown. At the beginning of the 1970s, encouraging results in terms of overall survival were reported and were attributed to the second-look strategy, although controversial results have subsequently been published without any further findings supporting additional surgery without the evidence of recurrent disease [11,12,13].

The role of carcinoembryonic antigen (CEA) as a prognostic recurrence marker was explored in a randomized trial during the 1980s and 1990s without demonstrating any additional benefit in survival [14]. With regard to pulmonary metastasectomy, the cornerstone is still represented by the International Registry of Lung Metastases, including 5206 metastasectomies from 18 centers, classified into epithelial cancers, germ cell, melanoma and sarcoma. Pulmonary metastasectomy from CRC was reported to have the best long term results [15]. Similar results had been previously reported by the Memorial Sloan Kettering Cancer Centre, first introducing the concept of CRC pulmonary metastasectomy into daily clinical practice [16,17].

## 4. State of the Art

According to the European Society of Medical Oncology (ESMO) Clinical Practice Guideline for diagnosis, treatment and follow-up of metastatic CRC, the definition of oligometastatic disease is related to the following conditions: usually one to five metastases, occasionally more when radical resection is feasible; up to two metastatic districts; primary tumor is controlled, ideally radically resected; all detected metastases should be safely treated by local treatments [18]. This definition relies on imaging findings and clinical evaluation, without taking into consideration—at the moment—biological features; however, further aspects should be carefully considered before proposing local treatments to oligometastatic CRC patients. Number, volume and sites of metastases, status of primary tumor, disease free interval, previous therapeutic approaches and their results, as well as global prognosis, contribute to the best choice in terms of local treatments [19]. 

In the vast majority of cases, the first therapeutic approach to metastatic CRC is a systemic induction treatment, whose grade of response or stabilization represents an effective predictor in terms of good prognosis, thus further supporting an additional loco-regional control by local treatments in a sort of consolidative treatment. In some patients presenting with well-circumscribed metastatic diffusion and good clinical conditions, or on the other hand, when patients do not tolerate systemic treatments, upfront local treatments are considered as a standard of care [18]. Local control of limited metastatic disease can be taken into consideration, even in the case of oligo-progressive disease, a clinical scenario represented by minimal recurrence or absence of response in patients undergoing systemic therapy: in this setting, the main purpose of local treatments is to eradicate cell clones not responding to systemic therapy, thus allowing systemic therapy to continue more effectively. Furthermore, the effectiveness of a local regional rescue treatment in oligo-progressive diseases is directly proportional to time to progression: the longer the pain-free survival (PFS), the greater the possibility of benefit from loco-regional treatment. Local treatment of limited metastatic CRC disease—involving one or two organs, most commonly liver and lung—may result in a curative approach, attaining long-term survival and cure in 20–45% of cases receiving a radical resection by surgical approach or complete disease ablation by thermoablation (TA) or stereotactic radiotherapy (SRS) [20,21]. Given the lack of randomized controlled trials comparing surgical resection with other local treatments such as TA/SRS, surgical resection remains the gold-standard approach for resectable oligo-metastatic disease in fit patients; on the other hand, TA/SRS can be taken into consideration in the case of limited and small metastases in patients unfit for surgery [18,22,23,24].

In some cases of limited metastatic spreading to the lung, imaging surveillance, also known as the wait and see approach, rather than local treatment control has been proposed [25]. However, the only randomized clinical trial comparing pulmonary metastasectomy versus continued active monitoring in colorectal cancer (PulMiCC Trial) was prematurely stopped because of poor and slow time recruitment; in addition, the small number of enrolled patients made it impossible to properly respond to the trial question, considering the substantial overlap in the confidence intervals in the proportions still alive at all time points. Nevertheless, a 5-year absolute survival benefit has been observed with surgical resection of about 35–40% versus less than 5% in controls. In addition, an estimated survival of 38% for resected patients versus 29% of non-operated patients has been reported [26]. These findings, although not conclusive, seem to be in favor of pulmonary metastasectomy, although the best option for each patient should always be carefully evaluated by a multidisciplinary team (MDT) approach [27]. Surgical resection should be able to provide radical excision of metastatic disease and should always be taken into consideration, although in the wider clinical contest of prognostic data and technical complexity of the required resection. CRC metastases can be locally treated by non-surgical approaches, in particular when localized in the liver: the COLLISION trial, a phase III single-blind prospective randomized controlled trial comparing liver surgical resection and thermal ablation, proved that thermal ablation is not inferior to surgical resection in treating lesions ≤3 cm [28]. With regard to the lung, there is no robust evidence comparing radiofrequency ablation (RFA) and microwave ablation (MWA) with surgical resection, while high conformal hypo-fractionated SBRT is considered a treatment option, although it is yet unclear which patients benefit most [18]. Local treatment of metastatic sites in patients with extensive disease aims to improve long-term survival and extend progression-free survival as part of a multimodality approach, but rarely represents a curative approach [29] (Table 1). 

## 5. Classification of Lung Metastases from CRC

Lung metastases are classified—on the basis of time between CRC diagnosis and pulmonary metastasis appearance—into synchronous or metachronous metastasis: the first is diagnosed at the time of the diagnostic workup for CRC while the second is found after the diagnostic workup. Moreover, lung metastases can be defined as initial metastases, when the lung is the site of the first distal metastases, or non-initial metastases, when pulmonary lesions appear after metastases to other organs. Depending on whether the lung is the only affected organ or metastatic diffusion involves extrapulmonary districts, lung metastases are defined as isolated or non-isolated. Among patients diagnosed with initial lung metastases, isolated lung metastases have been observed in 38–45% of cases, of which only about one third are eligible for radical pulmonary metastasectomy [30,31,32].

Risk factors suggesting pulmonary metastases in CRC are as follows: patient older than 70, bilateral pulmonary nodules, development of lung nodules after the diagnosis of CRC (metachronous lung nodules), pleural effusion of suspected pleural lesions, primary tumor localized in the middle or lower rectum, advanced-stage CRC presenting vascular invasion, N+ disease, higher preoperative carcinoembryonic antigen (CEA) levels, CRC presenting KRAS mutation and synchronous or metachronous extrapulmonary metastases [33]. In the era of more detailed imaging techniques, the presence of synchronous lung solitary nodules should be approached with careful MDT supervision: the possibility of surgical exploration or invasive techniques to obtain a histologic sample should be mandated.

## 6. The Role of Surgery

Among patients diagnosed with pulmonary metastases from CRC, about 9–12% are eligible for local treatments with radical intent, including surgical resection, SRS or ablation therapy [30]. Due to the lack of randomized controlled trials, there is no definitive evidence about the optimal approach, although surgical resection is considered the most effective therapeutic option in this clinical scenario [32]. In fact, a 5-year survival rate ranging from 35 to 70% has been reported after surgical resection of metastatic lung lesions, while the 5-year survival rate for patients receiving only a systemic approach is about 20% [34,35]. In the light of these data, an aggressive surgical approach is recommended for patients eligible for pulmonary metastasectomy, and other local treatments can be taken into consideration in the case of unresectable disease due to the global volume of lung metastatic disease (number and locations), cardiopulmonary function and patient willingness [36]. 

## 7. Principles of Surgical Resection

The most frequent procedures for treating pulmonary metastases are parenchyma-sparing sublobar resections such as wedge resections, segmentectomies and lung tumorectomies. In very selected cases, standard pulmonary lobectomy could be required because of metastases volume, number or localization within the lobe. On the contrary, more extensive resections, although described in the past literature, should be avoided [37]. Less extensive resections should, in any case, be preferred, not only to minimize cardiopulmonary stress but also taking into consideration the possibility of further metachronous resection during the future clinical history of the patient. When preoperative imaging workout—by computed tomography and positron emission tomography—does not disclose pathological mediastinal or hilar lymph nodes, lymph node dissection can be skipped; on the contrary, in cases of suspected lymph node involvement, lymph node sampling or biopsy should be considered during surgery [38]. It has been observed that N2 and N1 node involvement in patients receiving pulmonary metastasectomy has a significant negative impact on survival. As a clinical consequence, preoperatively confirmed nodal involvement is a relative contraindication for surgical resection. Moreover, it has been reported that at least 13% of patients receiving lung metastasectomy present pathological lymph node metastases that were not shown by preoperative workup; as a consequence, more accurate and sensitive preoperative staging procedures are required [38]. In the case of resectable pulmonary metastases, perioperative treatments can effectively contribute to achieving radical excision and decreasing the chance of postoperative recurrence; moreover, induction treatments contribute to clearly showing the biological behavior of the lesions, thus allowing a more appropriate patient selection. At the moment, there is no unequivocal interpretation of resectable pulmonary metastases or at least potentially resectable pulmonary metastases. In the vast majority of cases, unresectability is due to the wide dissemination of nodules within lung parenchyma, but other options include centrally located lesions very close to hilar structures or high-volume lesions—often more than one—requiring major lung parenchyma sacrifice in order to achieve local radicality. Nevertheless, it should be clearly emphasized that some of these metastases might benefit from induction treatments which might result in disease reduction, thus allowing radical resection. Moreover, performance status and the risk of developing significant postoperative complications after lung resection in this cohort of patients should be taken into consideration [39].

Given that pulmonary metastases show the best prognosis among all CRC metastases, when other sites of metastases are involved, these other distal metastases should be considered the leading factor when deciding the therapy. When occurring with liver metastases—which represent the most frequent scenario—the therapeutic approach should be tailored to lung and liver resectability: if both sites are amenable for local radical treatment, given that radical local treatment of the primary tumor has been accomplished, lung metastases and liver metastases should be treated in stages. Moreover, six months of systemic perioperative treatment before and after local treatment should be administered [32]. If lung metastases are resectable but liver metastases are not, systemic therapy should be administered without proceeding with local treatments [40]. If lung metastases are not resectable but liver metastases are resectable, selective radical local treatment for liver metastases can be performed—based on the effective systemic treatment administered—while lung metastases should not be locally approached [41]. When both lung and liver metastases are not resectable, only systemic therapy should be administered [32]. When more than two sites of metastases are detected in addition to pulmonary metastases, then this scenario is not judged as oligometastatic disease, and systemic palliative therapy should therefore be considered the best therapeutic approach [32]. Nowadays, although chemotherapy—in combination with targeted therapy—is not judged to be able to switch initially unresectable metastatic disease into a resectable one, including pulmonary metastases, a limited number of patients might disclose a successful shift from unresectable to resectable disease; each patient suffering from metastatic CRC should therefore undergo careful evaluation by the MDT. In the case of single pulmonary metastasis, ablation therapy (radiofrequency or thermoablation) should be taken into consideration as a first approach when the target lesion is situated in the peripheral part of the lung; on the contrary, when the target lesion is located in the hilar region or very close to blood vessels, SRS should be considered as the first approach. When the lesion to be treated is placed in the middle part of the lung, both ablation techniques and SRS could be taken into consideration, depending on the available devices and experience of the center [36]. It has recently been demonstrated by Antonoff et al. that not all CRC patients receive pulmonary metastasectomy. In fact, older patients, patients treated closer to their home and those cured at low-volume centers less frequently received pulmonary metastasectomy for lung-limited CRC after curative resection of their primary tumor. In addition, these patients disclosed a worse overall survival when compared to those submitted to lung metastasectomy, thus highlighting social disparities still present in cancer care [42]. It has been reported that recurrence after pulmonary metastasectomy can reach 72% [43], among which a local recurrence rate is reported in about 50% of cases [44]. Given the high incidence of local recurrence after pulmonary metastasectomy, it is vital for postoperative recurrence to be promptly recognized in order to maximize survival benefit [45]. In fact, early diagnosis of local recurrence allows effective treatment options such as re-do pulmonary metastasectomy, providing significant survival outcomes [46]. A combination of clinical and genomic factors might significantly condition post-metastasectomy recurrence, thus being identified as prognostic factors suggesting a stricter postoperative follow-up in some patients [47,48].

Deboever et al. demonstrated that patients exhibiting KRAS or TP53 mutations are more likely to develop local recurrence after pulmonary metastasectomy and should therefore receive more frequent imaging follow-up during the early post-operative period [47]. This would provide an earlier diagnosis of recurrent lung nodules, thus allowing re-do metastasectomy—whenever possible—and providing a 5-year overall survival of up to 76.9%. Moreover, it has been shown that radiomics—integrated with pathological data—can effectively predict both disease-free and overall survival [47,48,49,50]. Liquid biopsy, together with circulating tumor cells, extracellular vesicle microRNAs and cell-free DNA, may represent an effective mix of proper methods to anticipate disease recurrence [51,52,53,54]. Ziranu et al. recently reported a clinical score for colorectal cancer patients with lung-limited metastases undergoing surgical resection defined as the “meta-lung score”: they retrospectively reviewed 260 consecutive CRC patients presenting oligometastatic lung disease. Factors significantly associated with poor prognosis were as follows: altered baseline carcino-embryonic antigen (CEA) levels, disease free interval (DFI) less than or equal to 12 months, pulmonary nodules larger than 2 cm (*p* = 0.0187), multiple resectable metastases and metastatic lymph node status of the primary tumor. These five clinical variables were chosen as tools for developing a clinical risk score by assigning one point for each variable, thus creating a resulting score ranging from 0 to 1 point. The 5-year survival rate in patients scoring 0 points was 88%, while no patients scoring 1 were still alive at 2 years. Moreover—although not inserted in the meta-lung score—the BRAF mutation was confirmed to be associated with a poor prognosis, while adjuvant chemotherapy did not add any significant benefit in OS [55].

With regard to surgical approach, thoracotomy is suggested when multiple small nodules have been disclosed by CT scan to allow a more precise manual palpation of the whole lung parenchyma. On the other hand, single and peripheral nodules can be safely resected by minimally invasive video-assisted or robot-assisted approaches. With regard to the extent of lung resection, parenchyma-sparing procedures should be preferred (nodulectomy, segmentectomy, wedge resections); lobectomy might be required in some cases, while nowadays, pneumonectomy should be avoided in this metastatic setting. It is important to provide adequate resection margins (>5 mm), which can be safely obtained by anatomical resection, segmentectomy or wedge resection; on the other hand, when performing tumorectomy, they cannot be properly assessed, and therefore this technique should be applied only in case of very small nodules; frozen sections for intraoperative check of resection margins should be always available in centers performing metastasectomy.

Depending on the volume and number of nodules to be resected, we would avoid very extended bilateral resections requiring major lung resections; moreover, in case of resectable bilateral lesions, we would recommend two-step thoractomic stage resection to avoid or minimize major post operative complications and severe impairment of pulmonary functions.

The possibility of integrating new artificial intelligence (AI) algorithms in clinical practice could lead to a better definition of residual prognosis after a lung CRC metastasis resection. Wang et al. recently reported the development of a combined nomogram model integrating the biological and genomics features of the CRC disease with radiomics imaging and immunoscore. They adopted a patch-level convolutional neural network training in a weakly supervised manner to perform whole-slide histopathological images survival analysis. Moreover, the authors used a synthetic minority oversampling technique and support vector machine classifier to detect radiomics features and build predictive signature. This proposal showed a good performance in predicting the efficacy of surgical procedures in terms of OS and DFS, emphasizing the role of a machine learning procedure in the field of prognostication [56].

## 8. Systemic Treatment for Advanced Disease

The main role of systemic therapy concerns the metastatic setting, which includes potentially resectable disease (conversion therapy) or non-resectable disease (palliative therapy). The selection of these two settings of patients is very important due to the different median survival rates [57]. Molecular characterization of the tumor, that is, baseline RAS/BRAF status-MMR status, seems to be associated with different metastatic profiles at baseline, as well as with resectability rates and survival rates [58]. Lung metastases, for example, are more common among RAS mutant patients. Regarding prognostic factors, the longest median OS after diagnosis of advanced disease (systemic therapy only) and in resected patients (conversion therapy) is associated with RAS/BRAF wild type status. In these patients, with left-side tumors, the preferred treatment option is chemotherapy doublets with anti-EGFR monoclonal antibodies. Cytotoxic triplets (FOLFOXIRI scheme) with or without bevacizumab also result in high response rates, particularly in RAS/BRAF mutant populations [18]. Conversion therapy has been evaluated in the FIRE-3 trial, in which upfront combination chemotherapy plus either anti-EGFR cetuximab or antiangiogenetic agent bevacizumab was administrated in KRAS wild type patients [59]. The surgical approach was retrospectively possible in 22% at baseline and 53% after best response; survival rates were, respectively, 51.3 months for resected patients after conversion therapy, 30.8 months for resectable but not resected patients and 18.6 months for unresectable disease. 

While the role of adjuvant chemotherapy (AC) in resected CRC is a validated practice for stage III and high-risk stage II, reducing recurrence and thereby contributing to longer overall survival (OS) [60], the efficacy of post-operative chemotherapy after curative resection for stage IV CRC has been debated, with conflicting results regarding benefits.

To date, there have been no reports of randomized clinical trials (RCT) that compared surgery alone to neoadjuvant chemotherapy or adjuvant chemotherapy combined with surgery in patients with pulmonary metastases alone who underwent complete resection. More data are available for systemic therapy after liver metastasectomy, though with conflicting results [61]. A recent metanalysis, specifically conducted on lung metastasectomy to CRC, took eight different trials into consideration with two major biases to be noted: first, the retrospective nature of all eight studies, and second, the different chemotherapeutic regimens used which vary between studies (intravenous 5-FU, TS-1, capecitabine, intravenous 5-FU plus oxaliplatin, intravenous 5-FU plus irinotecan, or molecular targeted agents) [62]. Even if benefits in OS and progression-free survival, relapse-free survival and disease-free survival are demonstrated in the final analysis, the study limitations do not allow strong recommendation of the use of adjuvant chemotherapy in clinical practice. If indicated, the preferred chemotherapeutic regimen remains intravenous or oral 5-FU plus oxaliplatin for six months [18]. Another interesting setting of the potential benefit of systemic treatment is the peri-operative setting: this is the case of technically resectable metastasis but unclear or negative prognostic factors, in which 6 months of perioperative FOLFOX has a good impact on survival parameters [63]. There is currently a lack of data regarding the impact on resectability for particular subsets of patients, such as immune checkpoint inhibitors in microsatellite instable (MSI) or mismatch repair (MMR) deficient colon cancer patients [64] or target agents in BRAF mutant patients [65], but it is likely that there will be some expansion in surgical approaches for advanced disease due to achieved overall response rate.

## 9. Conclusions

The oncologic cornerstones to take into consideration with lung metastasectomy are as follows: the primary cancer has been successfully resected or is amenable to radical resection; there are no further extrathoracic metastases which cannot be resected or properly controlled; the cardiopulmonary function and performance status of the patient are not contraindications for pulmonary surgical resection; there are no non-surgical alternative options with lower morbidity and similar oncologic outcomes. Factors significantly associated with poor prognosis are augmented CEA levels, DFI less than or equal to 12 months, lung nodules larger than 2 cm, multiple resectable metastases and positive lymph node status of the primary tumor. There are currently no completed RCTs suggesting the clear advantage of one treatment over another; radical resection, primary tumor and metastases biology and disease-free interval are thus independent prognostic factors which make it possible to define a cohort of patients which might significantly benefit from pulmonary metastasectomy.

## Figures and Tables

**Table 1:** The history of pulmonary metastasectomy for colorectal cancer.

Author	Year	Journal
Wangensteen OH [9]	1954	Surg Gynecol Obstet
Bacon HE [10]	1959	Dis Colon Rectum
Polk HC [11]	1971	Surgery
Ellis H [12]	1975	Br J Surg
Cochrane JP [13]	1980	Br Med J
Pastorino U [15]	1998	Chest Surg Clin N Am
Zampino [21]	2014	Ann Thorac Surg
Treasure T [26]	2019	Trial
Cervantes A [18]	2023	Ann Oncol
